# Pattern and process in the evolution of the sole dioecious member of Brassicaceae

**DOI:** 10.1186/2041-9139-5-42

**Published:** 2014-11-12

**Authors:** Valerie L Soza, Vietnam Le Huynh, Verónica S Di Stilio

**Affiliations:** Department of Biology, University of Washington, Box 351800, Seattle, WA 98195-1800 USA

**Keywords:** allopolyploidy, programmed cell death, dioecy, floral ontogeny, genome size, organ arrest, phylogenetic network, *PISTILLATA*, sex differentiation, unisexual flowers

## Abstract

**Background:**

*Lepidium sisymbrioides*, a polyploid New Zealand endemic, is the sole dioecious species in Brassicaceae and therefore the closest dioecious relative of the model plant *Arabidopsis thaliana.* The attractiveness of developing this system for future studies on the genetics of sex determination prompted us to investigate historical and developmental factors surrounding the evolution of its unisexual flowers. Our goal was to determine the evolutionary pattern of polyploidization of *L. sisymbrioides* and the timing and process of flower reproductive organ abortion. To that end, we used a combination of phylogenetics to place this species within the complex history of polyploidization events in *Lepidium* and histology to compare its floral ontogeny to that of its closest hermaphroditic relatives and to *A. thaliana*.

**Results:**

Using a nuclear locus (*PISTILLATA*), we reconstructed the gene tree among *Lepidium* taxa and applied a phylogenetic network analysis to identify ancestral genomes that contributed to the evolution of *L. sisymbrioides*. Combining this phylogenetic framework with cytological and genome size data, we estimated *L. sisymbrioides* as an allo-octoploid resulting from three hybridization events. Our investigations of flower development showed that unisexual flowers appear to abort reproductive organs by programmed cell death in female flowers and by developmental arrest in male flowers. This selective abortion occurs at the same floral developmental stage in both males and females, corresponding to *Arabidopsis* stage nine.

**Conclusions:**

Dioecy in Brassicaceae evolved once in *L. sisymbrioides* following several allopolyploidization events, by a process of selective abortion of reproductive organs at intermediate stages of flower development. Different developmental processes, but similar timing of abortions, affect male versus female flower development. An increased understanding of how and when reproductive organs abort in this species, combined with our estimates of ancestral genome contributions, ploidy and genome size, lay the foundation for future efforts to examine the genetic mechanisms involved in the evolution of unisexual flowers in the closest dioecious relative of the best studied model plant.

**Electronic supplementary material:**

The online version of this article (doi:10.1186/2041-9139-5-42) contains supplementary material, which is available to authorized users.

## Background

The family Brassicaceae contains approximately 338 genera and 3,709 species [1, 2] and includes the model plant *Arabidopsis thaliana*, which has a floral morphology representative of the vast majority of the family. Only 5% of genera within Brassicaceae show deviations from the basic floral plan of four sepals, four petals, six stamens (four medial and two lateral), and two fused carpels [3]. One of these genera that diverge from the basic Brassicaceae floral morphology, *Lepidium* (230 species [1]), is widely distributed in temperate and subtropical areas [4]. Early-diverging lineages in the genus comprise outcrossing diploid species from the Old World that exhibit the basic floral plan of the family, whereas derived lineages tend to be selfing allopolyploids from the New World, Australia, and New Zealand with reduced flowers (that is, fewer stamens and/or reduced petals) [5]. Among the latter, the New Zealand endemic *Lepidium sisymbrioides* is the only dioecious species in the whole Brassicaceae [4, 6–10]. Staminate flowers of *L. sisymbrioides* consist of four to six stamens and a reduced ovary [10, 11], whereas carpellate flowers have three to seven staminodes and a functional pistil with style and stigma [10]. Nonfunctional reproductive organs of unisexual flowers of *L. sisymbrioides* have been loosely described as ‘abortive’ [8, 11], but the exact timing and process of the abortions remain unknown.

Incongruence between phylogenetic trees using nuclear versus chloroplast DNA regions suggests reticulate evolution within the genus [12]. It appears that hybridization, followed by whole genome duplication, resulted in predominantly allopolyploid *Lepidium* species in the Americas, Australia, and New Zealand [5]. These hybridization events may have contributed to the reduced stamens and petals observed in these species [5], in which case dioecy could represent another example of organ reduction.

In angiosperms, dioecy often follows polyploidization, presumably due to chromosomal rearrangements that facilitate the evolution of sex chromosomes or the breakdown of gametophytic self-incompatibility, followed by inbreeding depression [13–15]. Correlations between island habitat and dioecy are also common, through selection for outcrossing in small, colonizing hermaphroditic populations, [16]. In fact, New Zealand taxa in general show a higher incidence of gender dimorphism compared to their continental sister taxa [17, 18]. Combined evidence for reticulate evolution and polyploidy in New Zealand *Lepidium*[5, 12] suggests that *L. sisymbrioides* may also be an allopolyploid. We therefore hypothesize that this species represents another case of dioecy evolving in an island species, following hybridization and polyploidization.

The lineages containing *Lepidium* and *Arabidopsis* diverged from each other relatively recently, approximately 35 million years ago (mya) [19]. *Lepidium sisymbrioides,* therefore, offers the potential to uncover the genetic mechanisms involved in the evolution of unisexual flowers by being the closest dioecious relative to the most thoroughly investigated model plant. Determining the developmental stage and process of reproductive organ abortion should facilitate the identification of candidate genes involved in the evolution of unisexual flowers in this species as genes involved in sporogenesis and gametogenesis have been identified in *Arabidopsis*[20, 21]. Six developmental processes leading to reproductive organ abortion in unisexual flowers are recognized: cell death, programmed cell death, parenchymatization, arrest of development, change in timing of otherwise normal developmental events, and inviable pollen [22]. Identifying which of these processes contributes to the development of male and female flowers in *L. sisymbrioides*, as well as estimating this species’ genome size and ploidy history, will facilitate future efforts to uncover the genetic mechanisms involved in the evolution of dioecy.

The overall goal of this study was to investigate the pattern of polyploidization and the developmental processes underlying the evolution of separate sexes in *L. sisymbrioides*, the sole dioecious member of Brassicaceae. To that end, we 1) identified ancestral genomes within *L. sisymbrioides* and close relatives, 2) estimated ploidy and genome size for *L. sisymbrioides* and close relatives and 3) investigated the timing and process of organ abortion by comparing its floral development to that of its close hermaphroditic relatives and to *Arabidopsis thaliana*.

## Methods

### Sampling methods

Three subspecies of *L. sisymbrioides* were originally recognized: *kawarau* (Petrie) Thell., *matau* (Petrie) Thell., and *sisymbrioides*. All have been listed as nationally endangered because of a steep reduction in their distribution and abundance [23] and are difficult to sample. We sampled *L. sisymbrioides* subsp. *sisymbrioides* only because, despite reported habitat and morphological differences, all three subspecies are closely related [10].

In addition to published DNA sequences of *Lepidium* available from GenBank (Appendix 1), we sampled *L. sisymbrioides* subsp. *sisymbrioides* and three hermaphroditic close relatives endemic to New Zealand, *L. kirkii, L. naufragorum* and *L. tenuicaule*[9, 10, 12, 24, 25]. We sampled *L. kirkii* from an herbarium specimen (Appendix 1) and the remaining three species from cultivated accessions at the University of Washington (UW) greenhouse from wild-collected seed provided by P. Heenan (Landcare Research, Lincoln, New Zealand). Voucher specimens are listed in Appendix 1.

### Molecular methods

Because we were primarily interested in the polyploidization history of *L. sisymbrioides* and its close relatives (*L. kirkii*, *L. naufragorum,* and *L. tenuicaule*), we investigated reticulation events using the single-copy nuclear gene *PISTILLATA* (*PI*). *PI* had been previously used to detect reticulation among other *Lepidium* taxa [5] and therefore sequences were readily available (Appendix 1). Genomic DNA was extracted from one to two accessions for each of our four study species using the FastDNA Kit (MP Biomedicals, Solon, OH, USA) for cultivated accessions or following the protocol of Hughey *et al*. [26] for the herbarium specimen. We amplified and sequenced the first intron of *PI* using *PI*-ITF and *PI*-ITR primers [5]. Polymerase chain reaction conditions were 95°C for 2 min, followed by 35 cycles of 94°C for 30 s, 60°C for 1 min, and 72°C for 1 min, with a final extension step at 72°C for 5 min. Amplified DNA was purified using ExoSAP-IT (USB Corporation, Cleveland, OH, USA).

To distinguish among allelic variants, PCR products were cloned into the pCRII or pCR2.1 vector using the TA Cloning Kit (Invitrogen Corporation, Carlsbad, CA, USA). Plasmids were extracted using the FastPlasmid Mini Kit (5 Prime Inc., Gaithersburg, MD, USA). Three to 27 positive clones were sequenced per accession (UW Biochemistry DNA Sequencing Facility or GENEWIZ, Seattle, WA, USA) for a total of 20 to 35 clones per taxon.

### Phylogenetic analyses

*PISTILLATA* sequences were edited in Sequencher 4.9 (Gene Codes Corporation, Ann Arbor, MI, USA). We incorporated sequences we generated from our four study species plus available sequences from other taxa in GenBank to the entire alignment of the *PI* first intron, provided by J. L. Bowman [5]. We then aligned all sequences manually using MacClade 4.08 [27]. Ambiguously aligned regions were excluded from subsequent analyses. Our data set is available through TreeBASE (http://purl.org/phylo/treebase/phylows/study/TB2:S11886).

In order to detect whether PCR-mediated recombination had occurred among the *PI* copies within a species, we checked for recombination using RDP4 [28]. The *PI* alignment was analyzed by the automated exploratory recombination analysis, which employs eight different recombination methods: RDP [29], BootScan [30], GENECONV [31], MaxChi [32], Chimaera [33], SiScan [34], 3Seq [35], and LARD [36]. Analyses were run under the default general settings but as linear sequences and disentangling overlapping signals. The default settings for each method were used except for the following models: Felsenstein 1984 for BootScan and reversible process for LARD. Five recombinant sequences were identified and removed from the *PI* alignment before subsequent phylogenetic analyses.

We reconstructed the phylogeny for the *Lepidium PI* data set using Bayesian and likelihood analyses. For taxa that had multiple clonal *PI* sequences, we chose one clonal sequence from each monophyletic group of sequences representing a given taxon that was recovered in a 50% majority rule consensus tree from preliminary Bayesian analyses of all clones and that was representative of the majority of clones from a group. All other clonal sequences not forming monophyletic groups with other clones from the same taxon were included in the final analyses (Appendix 1).

For Bayesian and likelihood analyses, the model of evolution for the *PI* data set was determined by jModelTest 2.1 [37, 38]. The model selected under the Akaike Information Criterion [39] was TVM + I + Γ. We specified *L. phlebopetalum* and *L. perfoliatum* as outgroups, as previously identified in various studies [5, 9, 25, 40].

Bayesian analyses were conducted in MrBayes 3.2.2 [41, 42] via the CIPRES Science Gateway 3.3 [43]. We used default priors of no prior knowledge for the parameters of the model. Bayesian analyses were conducted with three independent Markov Chain Monte Carlo [44] analyses of 10 million generations each. Metropolis coupling for each analysis was conducted under the default settings. Convergence was determined when the average standard deviation of split frequencies remained less than 0.01. The first 10% of trees was discarded before convergence. The remaining trees from each run were pooled to construct a 50% majority rule consensus tree to obtain posterior probabilities (pp) and visualized with FigTree 1.4 [45].

Likelihood analyses were conducted in GARLI 2.0 [46]. Analyses were run under the default settings and included five search replicates to determine the maximum likelihood tree. To assess the reliability of clades in the resulting likelihood tree, we conducted 1,000 nonparametric bootstrap (bs) replicates [47] in GARLI. Bootstrap replicates were conducted under the above settings, but included one search replicate and 10,000 generations as the first part of the termination condition. Bootstrap trees were summarized with SumTrees 3.3.1 [48] and visualized with FigTree.

Since multiple *PI* copies in polyploid *Lepidium* taxa had been previously ascribed to allopolypoidy [5], we wanted to identify potential hybridization events leading to the evolution of dioecy in *L. sisymbrioides*. To facilitate visualization of potential ancestral genomes that contributed to the evolution of *L. sisymbrioides* and its close relatives (*L. kirkii*, *L. naufragorum, L. tenuicaule*), we conducted network analyses using the 50% majority rule consensus tree from Bayesian analyses of the *PI* data set as a multilabeled tree (MUL tree). The MUL tree was imported into Dendroscope 3.2.10 [49] and transformed into a phylogenetic network using the Huber *et al*. [50] algorithm, which minimizes the number of hybridization nodes.

### Cytology

Chromosome counts were obtained from pollen mother cells (PMCs) from freshly collected floral buds from one to two cultivated accessions of *L. sisymbrioides* and *L. tenuicaule* using a modified protocol of Kato [51]*.* Floral buds were treated according to the protocol of Matsushita *et al*. [52] and Wright *et al*. [53], modified with an N_2_O treatment for 3 hours at 206 PSI and an enzyme digestion for 3 hours. PMCs were mounted in VECTASHIELD with DAPI (Vector Laboratories, Burlingame, CA, USA), observed and photographed using a Nikon Microphot-FX microscope (Nikon Instruments, Inc., Melville, NY, USA) and a Retiga 1300 monochrome camera (QImaging, Surrey, British Columbia, Canada).

### Genome size estimation

Three accessions from three species cultivated at the UW greenhouse (*L. naufragorum, L. sisymbrioides,* and *L. tenuicaule*) were analyzed to obtain relative holoploid genome size, expressed as a 1C-value as defined in [54]. Nuclei were extracted from fresh leaf tissue and combined with chicken erythrocyte nuclei (CEN singlets, BioSure, Grass Valley, CA, USA) before staining with propidium iodide and analyzed with a flow cytometer, as outlined in Davison *et al*. [55]. CEN, with a 1C-value of 1223 Mbp or 1.25 pg [56], were used as an internal calibration standard. Animal standards have been discouraged by some authors for plant studies because (1) they cannot account for the huge range of plant genome sizes, (2) their nuclei structure may be different from plant nuclei, and (3) their precise genome size is unknown [57]. However, for our purposes, the genome size of CEN falls within the range of *Lepidium* genome size estimates. Additionally, our goal was to produce relative holoploid genome size estimates, since absolute estimates are not feasible due to the lack of complete genome coverage in most model taxa because of repetitive regions in the genome [55, 57].

Samples were analyzed on a FACScan flow cytometer (Becton, Dickinson and Company, Franklin Lakes, NJ, USA) with FlowJo software (Tree Star, Ashland, OR, USA) at the UW Department of Immunology Cell Analysis Facility. The 2C median nuclear peak of propidium iodide fluorescence in *Lepidium* samples was compared to that of the CEN standard to estimate the 2C nuclear DNA content of *Lepidium* in Mbp, then converted to pg using the equation from Dolezel *et al*. [58].

### Floral development observations

Floral tissue of *L. naufragorum*, *L. sisymbrioides*, and *L. tenuicaule* was dissected and fixed overnight in formaldehyde-acetic acid-alcohol (FAA). Prior to scanning electron microscopy (SEM), tissue was dehydrated through an ethanol series (30 min each of 50%, 60%, 70%, 85%, 95% and 100%), critical-point dried, mounted, and sputter coated at the UW Department of Biology Imaging Facility. Observations were made with a JSM-840A scanning electron microscope (JEOL, Peabody, MA, USA).

The relative timing of floral organ initiation and growth in *Lepidium* taxa had been previously shown to be comparable to that of its model Brassicaceae relatives (that is, *Arabidopsis thaliana* and *Brassica napus*), with the exception of petal growth and stamen number [59, 60]. Therefore, floral developmental stages for our three *Lepidium* study species were designated by the 13 characterized stages of the closely related model *Arabidopsis thaliana*[20, 61, 62].

For histological observations, inflorescences were fixed in FAA, then dehydrated through an ethanol series ending in Citrisolv (Fisher Scientific, Kent, WA, USA), embedded in Paraplast Plus (McCormick Scientific, LLC, St. Louis, MO, USA), and sectioned (5 or 8 μm) according to the protocol of Kramer [63]. Slides were deparaffinized with CitriSolv, hydrated through an ethanol series, stained in 1% Safranin O for 24 hr [64] and counterstained with 0.5% Fast Green FCF for 30 sec or stained in 0.05% Toluidine Blue O in dH2O for 1 to 2 min, and dehydrated through an ethanol series ending in CitriSolv. Histological sections were mounted in Cytoseal™ 60 (Richard-Allan Scientific, Kalamazoo, MI, USA) and observed using a Leica TCS SP5 II laser scanning confocal microscope (Leica Microsystems Inc., Buffalo Grove, IL, USA) with an excitation of 488 nm and an emission of 500 to 560 nm for Safranin O and an excitation of 561 nm and an emission of 625 to 690 nm for Fast Green FCF or using a Leitz Orthoplan 2 microscope (Ernst Leitz, Midland, Ontario, Canada) and photographed with a MicroPublisher 3.3 Real-Time Viewing camera (QImaging, Surrey, British Columbia, Canada).

### TUNEL assays

We conducted TUNEL assays to determine whether programmed cell death (PCD) was occurring in aborted stamens from *L. sisymbrioides* female flowers. Paraffin-embedded tissue sections were prepared as outlined above from female *L. sisymbrioides* and male *L. sisymbrioides* and hermaphroditic *L. tenuicaule* for comparison. We used the DeadEnd Fluorometric TUNEL System (Promega Corporation, Madison, WI, USA) according to manufacturer’s instructions, including positive controls, and washed slides in PBS containing 0.1% Triton® X-100 and 5 mg/ml of BSA after terminating reactions to reduce background as recommended. Slides were mounted in VECTASHIELD with DAPI, except for negative control slides that were untreated and mounted in Cytoseal™ 60. Slides were observed using a Leica TCS SP5 II laser scanning confocal microscope using an excitation of 405 nm and an emission of 430 to 550 nm for DAPI and an excitation of 488 nm and an emission of 500 to 535 nm for fluorescein.

## Results

### *PISTILLATA*gene duplication history suggests allopolyploidy in *Lepidium sisymbrioides*and relatives

**Figure 1:**
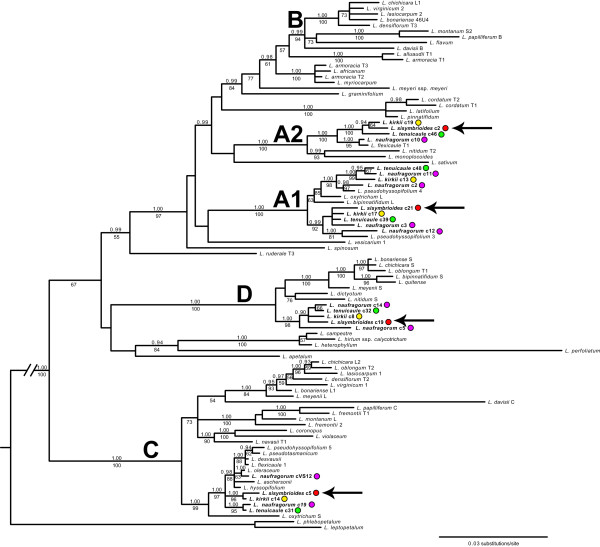
**Bayesian majority rule consensus tree of the first intron of the nuclear** ***PISTILLATA*** **(** ***PI*** **) gene in** ***Lepidium*** **.** Four *PI* clades previously identified from a number of taxa within the genus are denoted as **A-D** (after [5]). Clade ‘A’ is not monophyletic in our study, and new clades identified by our study are denoted as A1 to A2. Sequences from *Lepidium* taxa that were generated in our study are in bold and indicated by a colored dot: *L. kirkii* (yellow), *L. naufragorum* (purple), *L. sisymbrioides* (red), *L. tenuicaule* (green). Four *PI* copies from *L. sisymbrioides* are indicated by arrows. Posterior probabilities ≥0.90 and maximum likelihood bootstrap values >50% are displayed above and below branches, respectively.

In order to identify hybridization events leading to the evolution of the dioecious species *L. sisymbrioides* and its closest hermaphroditic relatives, *L. kirkii, L. naufragorum* and *L. tenuicaule*, we amplified and sequenced the first intron of the single-copy nuclear gene *PI* from these species and aligned them to other *Lepidium* sequences available in GenBank or unpublished (provided by J. L. Bowman; Appendix 1). Phylogenetic analyses recovered five, strongly supported clades (A1-D; pp ≥0.99, bs ≥94%; Figure 1) representing five major copies of the *PI* intron from American, Australian, and New Zealand (AANZ) taxa. Multiple copies of the *PI* intron within a taxon were previously suggested as representing multiple genomes from allopolyploid hybridization [5]. Clades A1 and A2 were denoted here because they had been previously recognized as a single clade ‘A’, but with low support [5]. In our analysis, these two clades are strongly supported as distinct (pp = 1.00, bs = 100%) and indicative of two separate genomes, as evidenced by sequences from our four study species in both clades (Figure 1, colored dots). Therefore, we found at least four distinct copies of the *PI* intron in *L. kirkii* [GenBank:KJ648155-KJ648159]*, L. naufragorum* [GenBank: JN119859-JN119860/JN119862/KJ648160-KJ648165], *L. sisymbrioides* [GenBank: KJ648166-KJ648169], and *L. tenuicaule* [GenBank:KJ648170-KJ648174], representing clades A1, A2, C, and D (Figure 1). In addition, multiple sequences of *L. kirkii, L. naufragorum*, *L. pseudohyssopifolium,* and/or *L. tenuicaule* within clades A1, C, and D (Figure 1) suggest that hybridization, gene duplication and/or allelic divergence are at play*.* None of the New Zealand taxa studied fell into the fifth clade B, which consists entirely of American taxa. Our results therefore suggest that all four New Zealand species are allopolyploids (and potentially allo-octoploids at minimum), originating from at least four divergent genomes (represented by clades A1, A2, C, and D).

**Figure 2:**
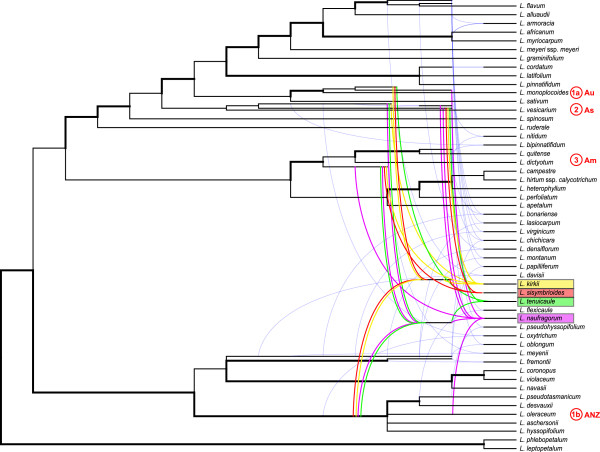
**Phylogenetic network from the Bayesian majority rule consensus tree of the first intron of the nuclear** ***PISTILLATA*** **(** ***PI*** **) gene in** ***Lepidium*** **.** The network is reconstructed using the Huber *et al*. [50] algorithm. Strongly supported branches from the Bayesian consensus tree, with posterior probabilities >0.90 and/or maximum likelihood bootstrap values >75%, are indicated in bold. Allopolyploids result from curved lines. Our four study species are highlighted by colored lines, and the other 17 non-focal allopolyploids are depicted by lighter, thinner blue lines. Ancestral genomes contributing to our study species *L. kirkii*, *L. naufragorum*, *L. sisymbrioides*, and *L. tenuicaule* are indicated in colors matching Figure 1: yellow, purple, red, and green lines, respectively. Four distinct ancestral genomes (red) contributing to *L. sisymbrioides* are numbered on the right (1a, 1b, 2 and 3) with their respective biogeographical origin: Am, America; As, Asia; Au, Australia; ANZ, Australia/New Zealand.

We further used the Bayesian majority rule consensus tree from the *PI* data set (Figure 1) to estimate a phylogenetic network to aid in the identification of hybridization nodes and potential ancestral genomes contributing to our study species (Figure 2). According to the network, our sampling includes 21 allopolyploid *Lepidium* taxa (Figure 2, tree branches originating from curved lines), which are confirmed polyploids from the literature [5] and this study (*L. sisymbrioides*, *L. tenuicaule*). The remaining 31 taxa in our analyses do not show evidence of reticulation, and this may be due to diploidy, autopolyploidy, or gene loss.

The evidence suggests that *Lepidium sisymbrioides* is derived from four distinct ancestral genomes (Figure 2, red lines): (1) a hybrid between (a) a descendant from the common ancestor of the *L. monoplocoides* group and (b) the common ancestor of the group that includes *L. pseudotasmanicum* and *L. hyssopifolium* (strong support), (2) a descendant from the common ancestor of the *L. vesicarium* group (low support), and (3) a descendant from the common ancestor of the *L. dictyotum* and *L. quitense* group (strong support). Biogeographically, the contribution of these genomes to *L. sisymbrioides* implies hybridization among Australian and New Zealand (ANZ) taxa (1a and 1b, above), followed by hybridization with American (3) and potentially (with low support) Asian (2) species. The other three New Zealand study species, *L. kirkii*, *L. naufragorum*, and *L. tenuicaule*, show contributions from four, five, and four distinct ancestral genomes, respectively (Figure 2). Of these three close hermaphroditic relatives, *L. sisymbrioides* shares the most reticulation history with *L. kirkii*, followed by *L. tenuicaule*, then *L. naufragorum* (Figure 2).

### Cytological observations reveal octoploidy in dioecious *L. sisymbrioides*and its hermaphroditic relative *L. tenuicaule*

In order to confirm our results from the *PI* data set, we conducted chromosome counts in PMCs. Seed of *L. kirkii* was not available, so its chromosome number remains unknown.

**Figure 3:**
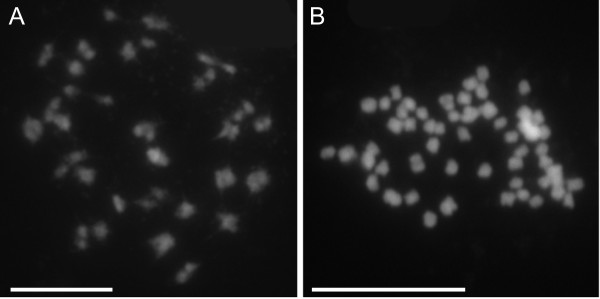
**Chromosome spreads from pollen mother cells of** ***Lepidium sisymbrioides*** **and** ***L. tenuicaule*** **. (A)** *L. sisymbrioides*: 32 pairs observed during late Prophase I of meiosis. **(B)** *L. tenuicaule*: approximately 64 chromosomes observed during early Prophase I of meiosis. Scale bar =100 μm.

Both *L. sisymbrioides* and *L. tenuicaule* had 2*n* = 64 chromosomes (Figure 3), corresponding to a ploidy of 8*x* (*x* = 8 is the base chromosome number for the genus [2, 4]). Octoploidy in these two species is consistent with having four distinct copies of the *PI* intron as shown by our phylogenetic and network analyses (A1, A2, C, D, Figures 1, 2). These four copies would therefore represent four distinct diploid genomes in these species’ history of hybridization and polyploidization events.

### Genome size estimations confirm ploidy estimates in *Lepidium sisymbrioides*and relatives

We examined holoploid genome size by calculating 1C-values for three of our New Zealand *Lepidium* study species to confirm our estimates of ploidy and to inform future genomic sequencing plans. *Lepidium sisymbrioides* and *L. tenuicaule* had similar holoploid genome sizes of 0.63 and 0.66 pg, respectively, whereas *L. naufragorum*’s holoploid genome size was slightly over double that of the other two species at 1.41 pg (Table 1). Material from which the holoploid genome size of *L. naufragorum* was obtained had published chromosome counts from the same population, indicating a ploidy of approximately 18*x*[65]. In conclusion, our holoploid genome size estimations are consistent with *L. sisymbrioides* and *L. tenuicaule* both being 8*x* and with *L. naufragorum* being 18*x*, more than double the ploidy of the former two species.

**Table 1:** **Mean holoploid genome size (1C-value) and ploidy estimates for three** ***Lepidium*** **species investigated**

Species	1C-value (Mbp +/- s.d.)	1C-value (pg)	Estimated ploidy level ^a^
*L. sisymbrioides*	621 +/- 7.84	0.635	**8x** ^**b**^
*L. tenuicaule*	645 +/- 29.41	0.660	**8x** ^**b**^
*L. naufragorum*	1379 +/- 61.56	1.410	18x^c^

^a^Ploidy estimates resulting from this study in bold.

^b^This study, estimated based on chromosome counts (Figure 3).

^c^[65].

### Female and male flowers of *Lepidium sisymbrioides*abort reproductive organs at comparable developmental stages but due to different processes

**Figure 4:**
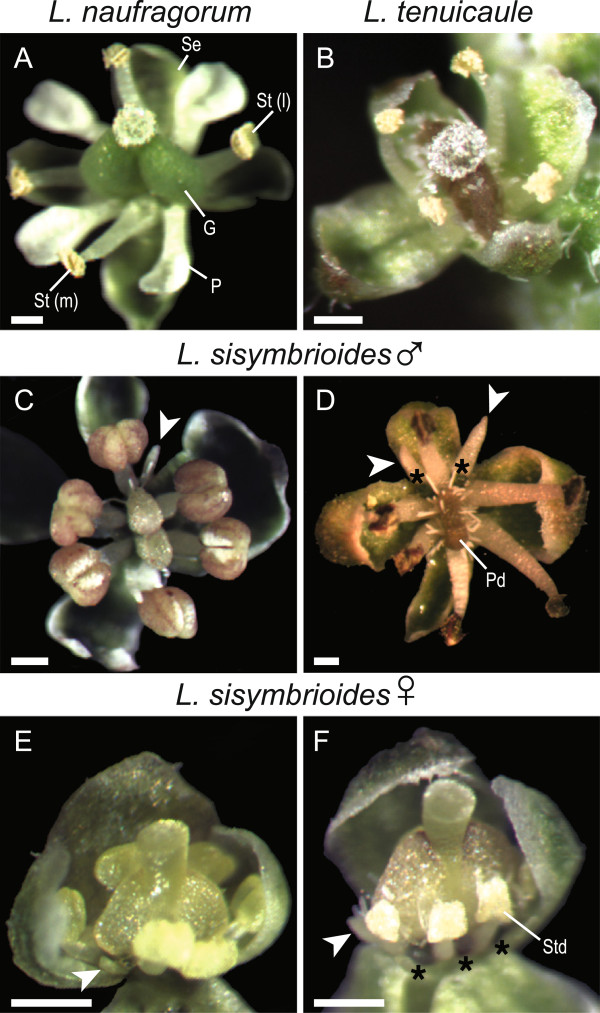
**Flower morphology of dioecious** ***Lepidium sisymbrioides*** **and two hermaphroditic relatives. (A)** *L. naufragorum*, hermaphroditic flower with full-sized petals, four stamens (two medial and two lateral), and a functional gynoecium. **(B)** *L. tenuicaule*, hermaphroditic flower with four medial stamens and a functional gynoecium (petals absent). **(C-F)** *L. sisymbrioides*. **(C)** Young male flower with reduced petals (one visible, arrowhead), six stamens (four medial and two lateral), and an aborted gynoecium. **(D)** Mature male flower with reduced petals (two visible, arrowheads), functional stamens, central pistillode, and interstaminal nectaries (asterisks). **(E)** Young female flower with reduced petals (one visible, arrowhead), six stamens (four medial and two lateral) slightly delayed in their development, and gynoecium. **(F)** Mature female flower with reduced petals (one visible, arrowhead), staminodes, functional gynoecium, and interstaminal nectaries (asterisks). Scale bar = 250 μm. G, gynoecium; P, petal; Pd, pistillode; Se, sepal; St (l), lateral stamen; St (m), medial stamen; Std, staminode.

In order to assess the developmental stage and process of abortion of reproductive organs in *L. sisymbrioides*, we examined floral morphology and ontogeny of this species in comparison to the two closest hermaphroditic relatives available, *L. naufragorum* and *L. tenuicaule*. Since floral developmental stages of hermaphroditic *Lepidium* species are comparable to the *A. thaliana* ontogenetic staging [20, 61, 62], we cross-referenced to this system for convenience and reproducibility. Flower morphology of *L. naufragorum* and *L. tenuicaule* differed from *A. thaliana* in petal size, number and arrangement of stamens, and ovule number. *Lepidium naufragorum* flowers had petals approximately as long as sepals and two lateral and two medial stamens (Figure 4A), whereas *L. tenuicaule* flowers had highly reduced petals, unnoticeable to the naked eye, and four medial stamens (Figure 4B). All *Lepidium* taxa produced a single ovule per locule.

**Figure 5:**
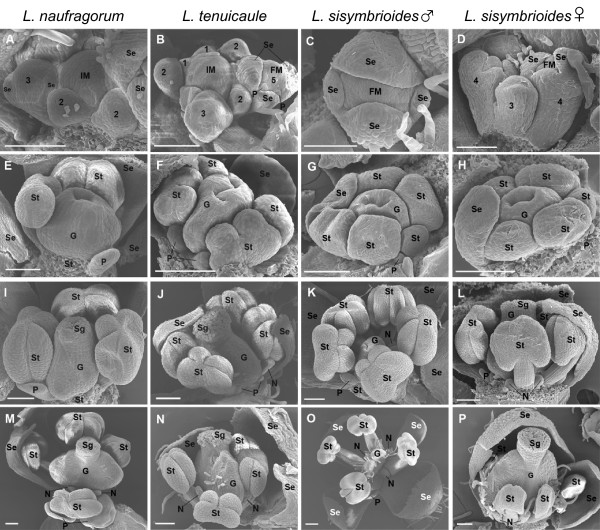
**Comparative flower ontogeny of dioecious** ***Lepidium sisymbrioides*** **and two hermaphroditic relatives by scanning electron microscopy. (A, E, I, M)** *L. naufragorum*. **(B, F, J, N)** *L. tenuicaule*. **(C, G, K, O)** Staminate *L. sisymbrioides.* **(D, H, L, P)** Carpellate *L. sisymbrioides*. In some panels, organs have been removed. Floral developmental stages noted below follow *Arabidopsis thaliana* stages [20, 61, 62]. **(A-D)** Initiating floral primordia within an inflorescence with sepals beginning to enclose the floral meristem, numbered 1 to 5 according to floral developmental stage. **(E-H)** Petal, stamen, and gynoecium primordia develop, stages 6 to 8. **(I-L)** Locules of the androecium have differentiated; gynoecium fuses and begins lateral expansion of carpels and stigmatic papillae begin to develop in I-J and L, stages 10 to 11, but arrest in K. **(M-P)** Stigma differentiates from subtending style in M-N and P, stage 12, but arrests in O; stamen filaments expand in M-O, but remain short in P; petals expand in M, but remain aborted in N-P. Scale bar =100 μm in A-N, P; 200 μm in O. FM, floral meristem; G, gynoecium; IM, inflorescence meristem; N, nectary; P, petal; Se, sepal; Sg, stigmatic papillae; St, stamen.

In contrast to *L. naufragorum* and *L. tenuicaule*, both male and female flowers of *L. sisymbrioides* generally exhibited six stamens (two lateral and four medial; Figure 4C-F) with a few exceptions where only four medial (Figure 5O) or five stamens were found [see Additional file 1]. Petals were reduced (that is, shorter than sepals; Figure 4C-F, arrowheads), and four to six nectaries were present among the stamen filaments in both sexes (Figure 4D, F, asterisks; [see Additional file 1]). In staminate flowers, the gynoecium arrested its development at intermediate stages, after differentiation of the anther locules (Figure 4C) and remained as a pistillode while stamens expanded normally (Figure 4D), as in *L. naufragorum* and *L. tenuicaule* (Figure 4A-B). In young carpellate flowers, stamens and carpels looked normal (Figure 4E). In later stages, however, stamen development was visibly arrested resulting in staminodia, whereas the gynoecium developed normally (Figure 4F) as in *L. naufragorum* and *L. tenuicaule* (Figure 4A-B). From these morphological observations, both carpels and stamens from male and female flowers of *L. sisymbrioides*, respectively, appeared to abort at intermediate stages of flower development.

SEM of flower development in all three species showed flower meristems that initiated from the inflorescence meristem in a similar fashion to *Arabidopsis* (Figure 5A-B, D, *Arabidopsis* stages 1 to 2). As expected, sepal primordia developed first (Figure 5A-D, stages 3 to 4), followed by petals (Figure 5B), then presumably stamen and gynoecium primordia. Stamen filaments and anther locules differentiated within the androecium and the gynoecium developed as a tube through postgenital fusion of two carpels (Figure 5E-F, stages 7 to 8). Subsequently, *L. naufragorum* started to show more petal expansion than the other two species (compare Figure 5E-H). The gynoecial tube then closed at completion of postgenital fusion and began to differentiate a stigma with papillae (Figure 5I-J, L, stage 11). In staminate flowers of *L. sisymbrioides*, after filaments and anther locules of the androecium had differentiated from one another, the gynoecium was arrested in its development (Figure 5K). The carpels of functional gynoecia expanded laterally, elongating and reaching full maturity with a clearly differentiated style and stigma (Figure 5M-N, P, stage 12). In *L. sisymbrioides* staminate flowers, the gynoecium remained aborted at maturity (Figure 5O) in comparison to functional gynoecia described above. Stamen filaments continued to elongate (Figure 5M-O, stage 12), except in carpellate flowers of *L. sisymbrioides* where they remained much shorter than the gynoecium (Figure 5P). In *L. naufragorum*, the only species with noticeable petals when mature, petals continued to expand, reaching the length of stamens (Figure 5M). In the other two species, petal primordia were initiated (Figure 5F-H) but never expanded, remaining small throughout development (Figure 5J-L) and not visible at maturity (Figure 5N-P).

**Figure 6:**
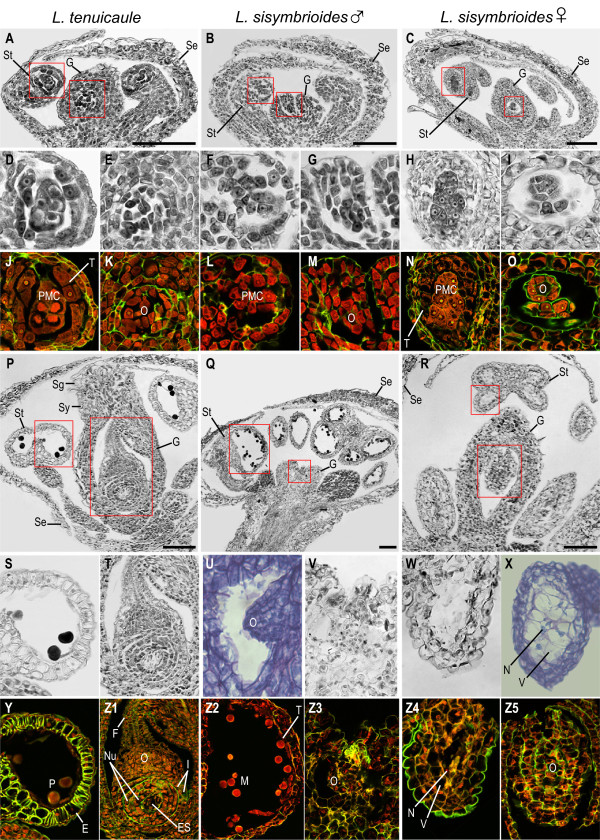
**Histological sections of hermaphroditic and dioecious** ***Lepidium*** **species.** Floral developmental stages noted below follow *Arabidopsis thaliana* stages [20, 61, 62]. **(A-C)** Bright field images of flowers at the pre-meiotic stage of microsporogenesis, stage 9, area outlined in red magnified below; from left to right, **(A)** hermaphroditic *L. tenuicaule*, **(B)** staminate *L. sisymbrioides,* and **(C)** carpellate *L. sisymbrioides*. **(D, F, H)** Bright field images of anther from respective flower above. **(E, G, I)** Bright field images of ovule from respective flower above. **(J, L, N)** Confocal microscopy of anther from respective flower above. **(K, M, O)** Confocal microscopy of ovule from respective flower above. **(P-R)** Bright field images of flowers at the microgametogenesis stage, stages 11 to 13, area outlined in red magnified below; from left to right, **(P)** hermaphroditic *L. tenuicaule*, **(Q)** staminate *L. sisymbrioides,* and **(R)** carpellate *L. sisymbrioides*. **(S, W)** Bright field images of anther from respective flower above. **(U)** Bright field images of ovule from male flower of *L. sisymbrioides* at stage 13 from Additional file 2. **(T, V)** Bright field images of ovule from respective flower above. **(X)** Bright field images of anther from female flower of *L. sisymbrioides* at stage 12 from Additional file 2. **(Y, Z2, Z4)** Confocal microscopy of anther from respective flower above. **(Z1, Z3, Z5)** Confocal microscopy of ovule from respective flower above. **(A-T, V-W, Y-Z5)** Stained with Safranin O and Fast Green FCF, **(U, X)** stained with Toluidine Blue O. Scale bar = 100 μm. E, endothecium; ES, embryo sac; F, funiculus; G, gynoecium; I, integuments; M, microspores; N, nucleus; O, ovule; P, pollen; PMC, pollen mother cells; Se, sepal; Sg, stigmatic papillae; St, stamen; Sy, style; T, tapetum; V, vacuolated cell.

Histological sections were performed to further investigate the anatomical development of stamens and carpels. *Lepidium naufragorum* [see Additional file 2] and *L. tenuicaule* revealed comparable reproductive organ development with no evidence of loss of organ function. Therefore, only data from *L. tenuicaule* is compared here against *L. sisymbrioides* (Figure 6).

In hermaphroditic flowers of *L. tenuicaule*, after stamen filaments and anther locules differentiated (Figure 6A), anthers consisted of PMCs, tapetum and two outer anther wall layers (middle layer and endothecium; Figure 6D, J, stage 9) and ovules began to develop in gynoecia (Figure 6E, K, stage 9). After meiosis of PMCs, anther wall layers degenerated, microspores underwent mitosis, and integuments enclosed the ovule [see Additional file 2, stage 12]. Subsequently, the androecium and gynoecium matured (Figure 6P). At this stage, the stamen filaments elongated (Figure 6P) and pollen sacs were composed of a single endothecium layer with secondary wall thickenings (Figure 6S, Y, stage 13). Pollen grains could be visualized with evident exine and the tapetum had degraded (Figure 6S, Y, stage 13). By this stage, the gynoecium had fused, elongated, and differentiated a style and stigma (Figure 6P, stage 13), and ovules had differentiated within each carpel (Figure 6T, Z1). Apical ovules consisted of an elongated funiculus and an embryo sac, surrounded by the nucellus and two integuments (Figure 6T, Z1, stage 13).

In staminate flowers of *L. sisymbrioides,* histological sections revealed that after initiation of the gynoecium (Figure 6B), sporogenous tissue (PMCs) was present in stamen locules (Figure 6F, L) and ovules had been initiated (Figure 6G, M, [see Additional file 2, stage 9]). However at later stages (Figure 6Q, stage 12; [see Additional file 2, stage 11]), as microspores matured within the anthers and the tapetum degenerated (Figure 6 Z2), the gynoecium failed to elongate and differentiate a style and stigma, and ovules did not grow nor differentiate (Figure 6U-V, Z3, [see Additional file 2]). Since the gynoecium arrest occurs before microsporogenesis (the production of tetrads from PMCs, stage 9), which normally precedes megasporogenesis (stage 11) in *Arabidopsis*, we conclude that the process for the loss of gynoecium function in male flowers is the arrest of development at a pre-meiotic, intermediate stage (stage 9).

**Figure 7:**
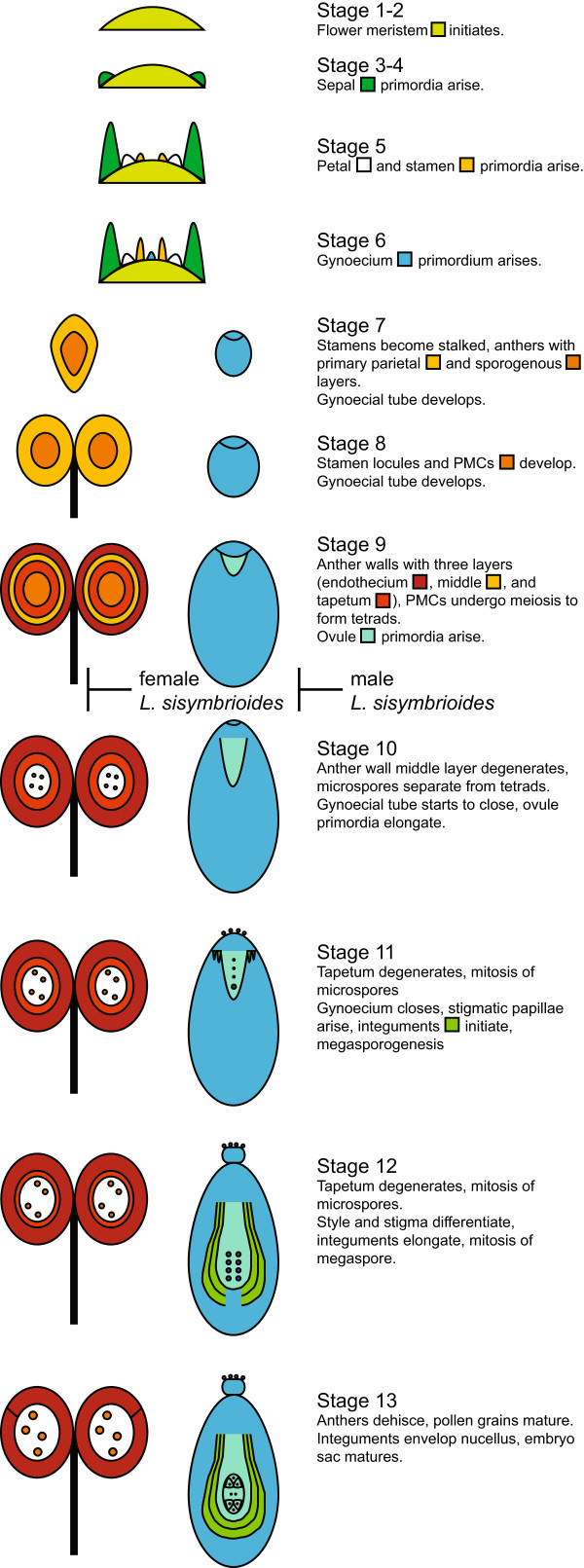
**Model for flower development in** ***Lepidium*** **study species, with proposed timing of reproductive organ abortion in dioecious** ***L. sisymbrioides*** **.** The model is informed by our SEM and histological observations (Figures 5, 6) and follows *Arabidopsis thaliana* stages, noted on the right [20, 61, 62]. Entire flower shown in stages 1 to 6, only one stamen (left) and one carpel of the bicarpellate gynoecium (right) shown in stages 7 to 13. A filament differentiates within the stamen in stage 8, and elongates in stage 9. **⊢** stage of abortion for female (left) and male (right) flowers of *L. sisymbrioides*.

In young carpellate flowers of *L. sisymbrioides* (Figure 6C), sporogenous tissue (PMCs) inside the stamen locules (Figure 6H, N) and ovule initiation (Figure 6I, O) were evident at the same stage as in hermaphroditic flowers (Figure 6A, D-E, J-K, stage 9). By the time the gynoecium closed and a stigma and ovule began to differentiate (Figure 6R, Z5, [see Additional file 2, stage 11]), vacuolated cells pervaded anthers and PMCs had degenerated (Figure 6W-X, Z4). Stamen filaments did not elongate and neither tetrads, microspores, nor pollen were produced; pollen sacs appeared shrunken, filled with vacuolated cells, and no endothecium layer developed (Figure 6W-X, Z4). Based on the above observations, we propose that the developmental process for loss of androecium function in female flowers is likely cell death, as evidenced by vacuolated cells (absence of stained cytoplasm) and nuclear degradation (Figure 6W-X, Z4) following the development of PMCs (Figure 6H, N). In conclusion, androecium abortion in female flowers occurs at a comparable pre-meiotic stage to gynoecium abortion in male flowers (stage 9) but due to different processes, that is, cell death versus developmental arrest, respectively. Figure 7 summarizes our SEM and histological observations on flower development in *Lepidium* study species (Figures 5, 6, [see Additional file 2]) in reference to described developmental stages from *A. thaliana*[20, 61, 62].

### Programmed cell death in anther walls is involved in the degradation of pollen mother cells of female *L. sisymbrioides*flowers

**Figure 8:**
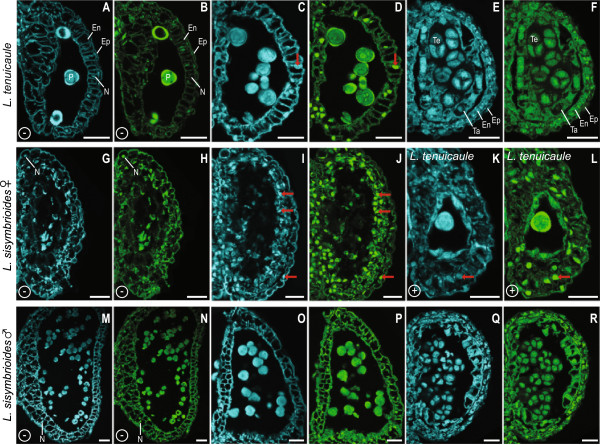
**Confocal microscopy of longitudinal sections of anther sacs from hermaphroditic, male and female flowers of** ***Lepidium*** **treated with TUNEL assay and controls. (A-F, K-L)** Hermaphroditic *L. tenuicaule.* **(G-J)** Carpellate *L. sisymbrioides*. **(M-R)** Staminate *L. sisymbrioides.* **(A, C, E, G, I, K, M, O, Q)** Anthers under DAPI excitation and emission. **(B, D, F, H, J, L, N, P, R)** Anthers under fluorescein excitation and emission. **(A-D, M-P)** Mature, functional anther sacs at stage 13. (G-J) Mature anther sacs, aborted at stage 9. **(E-F, Q-R)** Functional anther sacs forming tetrads, tapetum, and endothecium at stage 9. **(A-B, G-H, M-N)** Negative controls (-), anther sacs untreated: cell walls, nuclei, and pollen autofluoresce. **(C-F, I-J, O-R)** TUNEL assay. Red arrows point to an exemplary nucleus from each tissue layer that exhibits above-background fluorescein signal. **(K-L)** Positive control (+), anther sac treated with DNase I: nuclei in endothecium show strong fluorescein emission in L compared to B. Scale bar = 25 μm. En, endothecium; Ep, epidermis; N, nucleus; P, pollen; Ta, tapetum; Te, tetrads.

Because we were finding evidence of cell death in stamens of female *L. sisymbrioides*, we wanted to determine whether PCD could be responsible for this abortion of stamens. To look for evidence of PCD, as characterized by DNA fragmentation, we conducted TUNEL assays on histological sections of carpellate *L. sisymbrioides* and, for comparison, staminate *L. sisymbrioides* and *L. tenuicaule* flowers. The TUNEL assay attaches fluorescein to fragmented DNA, eliciting a green fluorescent signal in nuclei undergoing DNA degradation. *Lepidium* tissue autofluoresced in the absence of staining under both DAPI and fluorescein excitation and emission ranges: cell walls, chloroplasts, nuclei, and pollen grains showed background signal (compare Figure 8A-B to C-D, G-H to I-J, and M-N to O-P). This autofluorescence contributed additional histological evidence that cell death was occurring in stamens of female *L. sisymbrioides*, as evidenced by the absence or degradation of cell walls, nuclei, and pollen in the center of anther locules, where sporogenous tissue leading to pollen normally develops (compare Figure 8I-J to C-F and O-R). In spite of this autofluorescence, the use of negative and positive controls allowed us to observe strong, above-background, fluorescein signal in certain tissues at certain stages that indicate DNA degradation. For example, all nuclei in the endothecium of mature, functional anther sacs of *L. tenuicaule* at stage 13 showed a strong, above-background, fluorescein signal indicating PCD (compare Figure 8B to D, red arrow denotes one exemplary nucleus). More importantly, we observed strong, above-background, fluorescein signal in all nuclei throughout all anther wall layers of mature anthers from female *L. sisymbrioides* (compare Figure 8H to J, red arrows denote exemplary nuclei from each layer). This was taken as evidence that these nuclei are undergoing DNA degradation, as observed in positive controls (treated with DNase) showing higher than above-background signal (compare Figure 8B to L, red arrow denotes one exemplary nucleus). When comparing anthers from female *L. sisymbrioides* that abort at stage 9 to functional anthers from *L. tenuicaule* and male *L. sisymbrioides* at the same stage (compare Figure 8I-J to E-F and Q-R), it appeared that PCD in anther wall layers was contributing to the degradation of PMCs in carpellate *L. sisymbrioides* flowers, in which tapetum, endothecium, and tetrads do not develop as in functional anthers from *L. tenuicaule* and male *L. sisymbrioides* at the same stage. In summary, using the TUNEL assay as a proxy for PCD, we find evidence for PCD in the anther wall layers of carpellate *L. sisymbrioides* flowers, which likely contributes to the degradation of PMCs and abortion of anthers at stage 9.

## Discussion

*Lepidium sisymbrioides* is the sole dioecious member of Brassicaceae, and our phylogenetic analyses show that it is closely related to three other New Zealand hermaphroditic species: *L. kirkii, L. naufragorum* and *L. tenuicaule* (Figures 1, 2). Increased phylogenetic sampling of the *PI* first intron among AANZ taxa allows us to identify reticulation events leading to *L. sisymbrioides,* which resulted from three past hybridization events (Figure 2). Of the three close relatives, the shared reticulation history with *L. kirkii* is a novel finding. Molecular, cytological, and genome size analyses provide evidence that *L. sisymbrioides* is an allo-octoploid (Figures 1, 2 and 3, Table 1), with 64 chromosomes and an average holoploid genome size (1C-value) of 621 Mbp. By comparing the floral ontogeny of unisexual flowers in *L. sisymbrioides* to that of its close relatives and to *Arabidopsis thaliana*, we show that unisexual flowers in this species arose from selective abortion of reproductive organs at a similar floral developmental stage (Figure 7 stage 9) but by different processes in males and females. Differential abortion of the gynoecium in males appears to result from developmental arrest, while in females anther sterility results from programmed cell death (Figure 6, 8).

### Evolution of dioecy and unisexual flowers within Brassicaceae

The evolution of dioecy in Brassicaceae occurred only once in the genus *Lepidium*. We infer that in *L. sisymbrioides*, dioecy evolved from hermaphroditism via selective abortion of reproductive organs, as in type I flowers [66], as all other members of *Lepidium* are hermaphroditic. Our floral ontogeny observations confirmed that reproductive organs are initiated and differentially aborted at the same floral developmental stage in male and female flowers of *L. sisymbrioides*. The timing of abortion corresponds to *Arabidopsis* stage 9 (Figures 4, 5, 6 and 7), which is broadly considered an ‘intermediate’ stage of floral development [22], after primordia initiation but before meiosis. Our ontogeny shows that *L. sisymbrioides* is representative of the majority of angiosperms with type I unisexual flowers that selectively abort reproductive organs at significantly correlated developmental stages between the two sexes [22]. This evidence suggests that similar regulatory switch points underlie male and female developmental pathways as proposed by Diggle *et al*. [22] and comparable selective forces are at play in the two sexes. However, the developmental stage and process of reproductive organ abortion in unisexual flowers across angiosperms vary widely with different stages and processes occurring at equal frequencies [22].

With regard to the developmental process of organ abortion in *L. sisymbrioides*, while sporogenous tissue (PMCs) in stamen locules differentiates (Figure 6C, H, N), it quickly degenerates during development of carpellate flowers and becomes vacuolated with degraded nuclei (Figure 6R, W-X, Z4). Programmed cell death, which is involved throughout normal flower development [67], is primarily due to endogenous factors and is evidenced by cell death at a predictable time and location during tissue differentiation [68]. During normal flower development, the tapetum degenerates during microgametogenesis via PCD for proper microspore development and differentiation of pollen, [67]. Other studies have shown that premature tapetal degeneration can lead to male sterility [reviewed in 67]. Therefore, because we observe PCD in anther wall layers before microgametogenesis, this premature tapetal degeneration is likely leading to male sterility in *L. sisymbrioides* females (Figures 6R, W-X, Z4, 8I-J, [see Additional file 2]).

Two types of PCD occur in plants: autolytic and nonautolytic. The former generally occurs during normal plant development, whereas the latter occurs during plant-pathogen interactions [69, 70]. Moreover, since loss of cell walls and cytoplasm, nuclear condensation, and increase in vacuolar volume are characteristic of autolytic PCD [70], this type of cell death is also likely involved in the degeneration of PMCs in *L. sisymbrioides* females (Figures 6R, 8I-J, [see Additional file 2]).

In male flowers of *L. sisymbrioides*, on the other hand, the development of ovules and gynoecia is arrested shortly after initiation of ovule primordia. We found no evidence of cell death, parenchymatization, or change in timing of otherwise normal developmental events in arrested gynoecia. Ovule primordia remain evident in mature male flowers (Figure 6Q, U-V, Z3, Additional file 2). Therefore, of the six developmental processes reviewed in Diggle *et al*. [22], arrest of development best characterizes the abortion of the gynoecium in *L. sisymbrioides* males.

### Whole genome duplication events via hybridization in the evolution of dioecy in *Lepidium*

Two different copies of the *PI* first intron were previously identified among the ANZ taxa (clades A, C); only one copy (clade C) was strongly supported [5]. Our *PI* phylogeny recovered at least four divergent copies of the first intron in *L. sisymbrioides* and its close relatives (Figure 1), suggesting ancient allopolyploidization events, followed by divergence of *PI* alleles. Based on our phylogenetic network analyses, *L. sisymbrioides* has a history of three allopolyploidization events: hybridization (1) between an Australian and Australian or New Zealand species, (2) with an Asian species, and (3) with an American species (Figure 2). Our study provides new evidence of an additional genome within the ‘A’ clade of *PI*[5] and shows an additional *PI* copy in taxa from this clade (as in, *L. kirkii*, *L. naufragorum, L. sisymbrioides* and *L. tenuicaule;* A1-A2, Figure 1), which would be expected of four divergent genomes contributing to several allopolyploidization events in our study species. Based on our cytological and genome size estimates, *L. sisymbrioides* is an octoploid, which would require several whole genome duplications. Together with our *PI* data, this evidence suggests that *L. sisymbrioides* is an allo-octoploid composed of four different genomes.

Australian *Lepidium* appear to have undergone a rapid radiation during the Pliocene and Pleistocene, when the arid and cooler regions of the southeastern temperate biomes were expanding [71]. Previous studies suggested at least one dispersal event each from California and South Africa to Australia or New Zealand; most likely colonizing Australia first, with at least two subsequent dispersal events to New Zealand [12]. The majority of *Lepidium* species produce mucilaginous seeds that adhere to birds [4], which may have facilitated long-distance dispersal among the Americas, Australia, New Zealand and the Old World [72–75]. Our results suggest that a hybridization event occurred either within Australia or between an Australian and a New Zealand ancestor, followed by hybridization with an Asian colonist and an American colonist, resulting in the evolution of *L. sisymbrioides* (Figures 1, 2). Colonization by an Asian ancestor is not well supported by our data and conflicts with previous studies indicating colonization by an African ancestor [12]; this contradictory evidence could be due to the use of different nuclear DNA regions. In spite of this, our results confirm at least two dispersal events to Australia or New Zealand from the New World and Old World that resulted in allopolyploidization, but exact New and Old World ancestry is uncertain. Additionally, we infer a hybridization between ANZ taxa not previously suggested.

Our 1C-value estimates for *L. sisymbrioides* and *L. tenuicaule* fall within the reported range for the family (0.15 to 2.43 pg [76–78]). *Lepidium naufragorum* lies outside the high end of the range, consistent with it being highly polyploid (18x [65]). Even though the 1C-value of *L. sisymbrioides* (0.635 pg) is almost fourfold that of *Arabidopsis thaliana* (0.16 pg [79]), it is comparable to the size of other model plants such as rice (0.5 pg [80]), making it a likely candidate for whole genome sequencing. Genomic resources for this species would facilitate the investigation of sex determination and of the putative chromosomal rearrangements that contributed to the evolution of dioecy after polyploidization. As new technologies and approaches are being developed [81, 82], sequencing this octoploid will become more feasible in the near future.

## Conclusions

The developmental process leading to the evolution of dioecy in *Lepidium sisymbrioides* was placed in the broader context of the historical patterns conditioning the evolution of separate sexes in this unique dioecious relative of *Arabidopsis*. We have characterized the developmental stage and process of its unisexual flowers, paving the way for future studies aimed at unraveling the genetic basis underlying reproductive organ abortion. Having placed *L. sisymbrioides* in a phylogenetic context, determined its ploidy, hybridization history, and genome size, and compared it to *Arabidopsis thaliana* flower development will facilitate the investigation of the role of polyploidy and of potential candidate genes in the evolution of dioecy in Brassicaceae.

## Appendix 1

Voucher information and GenBank accessions for *Lepidium* taxa (Brassicaceae) sampled in this study. Voucher information provided only for taxa with sequences not downloaded from GenBank. Taxon, collector and collection number, origin, herbarium, *PISTILLATA* intron.

*Lepidium africanum* (Burm.f.) DC., AY114216; *Lepidium alluaudii* Maire, AY114221; *Lepidium apetalum* Willd., AY114217; *Lepidium armoracia* Fisch. & C.A. Mey., AY114218-AY114220; *Lepidium aschersonii* Thell., AY114222; *Lepidium bipinnatifidum* Desv., AY114223-AY114224; *Lepidium bonariense* L., AY114225-AY114227; *Lepidium campestre* (L.) R.Br., AY114228; *Lepidium chichicara* Desv., AY114229-AY114231; *Lepidium cordatum* Willd. ex Steven, AY114232-AY114233; *Lepidium coronopus* (L.) Al-Shehbaz, unvouchered, cultivated from wild-collected seed from Madrid, Spain (INIA seed accession 205-0261-68), JN119857; *Lepidium davisii* Rollins, FJ541471/FJ541473; *Lepidium densiflorum* Schrad., AY114235-AY114236; *Lepidium desvauxii* Thell., AY114237; *Lepidium dictyotum* A. Gray, AY114238; *Lepidium flavum* Torr., AY114239; *Lepidium flexicaule* Kirk, AY114240-AY114241; *Lepidium fremontii* S. Watson, AY114243-AY114244; *Lepidium graminifolium* L., AY114246; *Lepidium heterophyllum* Benth., AY114247; *Lepidium hirtum* (L.) Sm. ssp. *calycotrichum* (Kunze) Thell., AY114248; *Lepidium hyssopifolium* Desv., AY114249; *Lepidium kirkii* Petrie, P. Heenan s.n., cultivated from wild-collected seed from Galloway, New Zealand, CHR, KJ648155-KJ648159; *Lepidium lasiocarpum* Nutt., AY114250-AY114251; *Lepidium latifolium* L., AY114252; *Lepidium leptopetalum* F. Muell., AY114215; *Lepidium meyenii* Walp., AY114254-AY114255; *Lepidium meyeri* Claus ssp. *meyeri*, AY114269; *Lepidium monoplocoides* F. Muell., AY114256; *Lepidium montanum* Nutt., AY114257/AY114259; *Lepidium myriocarpum* Sond., AY114260; *Lepidium naufragorum* Garn.-Jones & D.A.Norton, V. Di Stilio 117, cultivated from wild-collected seed from Open Bay Islands, New Zealand, WTU, JN119859-JN119860/JN119862/KJ648160-KJ648165; *Lepidium navasii* (Pau) Al-Shehbaz, unvouchered, cultivated from wild-collected seed from Gádor, Spain (INIA seed accession 204-4472-76), JN119856; *Lepidium nitidum* Nutt., AY114261-AY114262; *Lepidium oblongum* Small, AY114263-AY114264; *Lepidium oleraceum* Sparrm., AY114265; *Lepidium oxytrichum* Sprague, AY114266-AY114267; *Lepidium papilliferum* (L.F. Hend.) A. Nelson & J.F. Macbr., FJ541451/FJ541488; *Lepidium perfoliatum* L., AY114268; *Lepidium phlebopetalum* (F. Mull.) F. Mull., AY114214; *Lepidium pinnatifidum* Ledeb., AY114270; *Lepidium pseudohyssopifolium* Hewson, AY114271-AY114274; *Lepidium pseudotasmanicum* Thell., AY114275; *Lepidium quitense* Turcz., AY114276; *Lepidium ruderale* L., AY114278; *Lepidium sativum* L., AY114279; *Lepidium sisymbrioides* Hook. f., V. Soza 1924, cultivated from wild-collected seed from Twizel, South Canterbury, New Zealand, WTU, KJ648166- KJ648169; *Lepidium spinosum* Ard., AY114280; *Lepidium tenuicaule* Kirk, V. Di Stilio 116, cultivated from wild-collected seed from Shag Point, New Zealand, WTU, KJ648170- KJ648174; *Lepidium vesicarium* L., AY114281; *Lepidium violaceum* (Munby) Al-Shehbaz, unvouchered, cultivated from wild-collected seed from N. Azrou, Morocco (INIA seed accession 206-4096-84), JN119858; *Lepidium virginicum* L., AY114285.

Notes: INIA = Instituto Nacional de Investigaciones Agrarias, Madrid, Spain.

## Electronic supplementary material

Additional file 1: **Staminate flowers of** ***Lepidium sisymbrioides.*** A) Young staminate flower of *L. sisymbrioides*, showing sepals (Se), five stamens (St), and aborted gynoecium (G). (B) Staminate flower of *L. sisymbrioides*, showing sepals (Se), six nectaries (N) among stamen (St) filaments, and aborted gynoecium (G). Scale bar = 0.25 mm. (ZIP 3 MB)

Additional file 2: **Histological sections of hermaphroditic and dioecious** ***Lepidium*** **species stained with Toluidine Blue O.** (A-C) Flower at the pre-meiotic stage of microsporogenesis, stage 9; from left to right, (A) hermaphroditic *L. naufragorum*, (B) staminate *L. sisymbrioides*, and (C) carpellate *L. sisymbrioides*. (D, F, H) Anther locule from respective flower above. (E, G, I) Ovule from respective flower above. (J-L) Flowers later on in development, at the microgametogenesis stage, stages 11–12; from left to right, (J) hermaphroditic *L. naufragorum*, (K) staminate *L. sisymbrioides*, and (L) carpellate *L. sisymbrioides*. (M, O, Q) Anther locule from respective flower above. (N, P, R) Ovule from respective flower above. (S-U) Mature flowers, stage 13; from left to right, (S) hermaphroditic *L. naufragorum*, (T) staminate *L. sisymbrioides*, and (U) carpellate *L. sisymbrioides*. (V, X, Z1) Anther locule from respective flower above. (W, Y, Z2) Ovule from respective flower above. Scale bar = 50 μm in A-C; 100 μm in J-U. E, endothecium; ES, embryo sac; G, gynoecium; I, integuments; M, microspores; N, nucleus; O, ovule; P, pollen; PMC, pollen mother cells; Se, sepals; Sg, stigmatic papillae; St, stamen; Sy = style; T, tapetum; V = vacuolated cell. (PDF 10 MB)

## Abbreviations

AANZ: American, Australian, and New Zealand
ANZ: Australian and New Zealand
bs: bootstrap
BSA: bovine serum albumin
CEN: chicken erythrocyte nuclei
DAPI: 4’,6-diamidino-2-phenylindole
FAA: formaldehyde-acetic acid-alcohol
Mbp: millions of base pairs
PBS: phosphate buffered solution
PCD: programmed cell death
pg: picogram
*PI*: *PISTILLATA*
PMCs: pollen mother cells
pp: posterior probabilities
SEM: scanning electron microscopy.
