# Supplementary material for: Pattern and process in the evolution of the sole dioecious member of Brassicaceae
Source: EvoDevo. 2014 Nov 12;5:42. doi: 10.1186/2041-9139-5-42 (PMC4407775; doi:10.1186/2041-9139-5-42)
Supplement: Supplementary file 2 — Additional file 2: Histological sections of hermaphroditic and dioecious Lepidium species stained with Toluidine Blue O. (A-C) Flower at the pre-meiotic stage of microsporogenesis, stage 9; from left to right, (A) hermaphroditic L. naufragorum, (B) staminate L. sisymbrioides, and (C) carpellate L. sisymbrioides. (D, F, H) Anther locule from respective flower above. (E, G, I) Ovule from respective flower above. (J-L) Flowers later on in development, at the microgametogenesis stage, stages 11–12; from left to right, (J) hermaphroditic L. naufragorum, (K) staminate L. sisymbrioides, and (L) carpellate L. sisymbrioides. (M, O, Q) Anther locule from respective flower above. (N, P, R) Ovule from respective flower above. (S-U) Mature flowers, stage 13; from left to right, (S) hermaphroditic L. naufragorum, (T) staminate L. sisymbrioides, and (U) carpellate L. sisymbrioides. (V, X, Z1) Anther locule from respective flower above. (W, Y, Z2) Ovule from respective flower above. Scale bar = 50 μm in A-C; 100 μm in J-U. E, endothecium; ES, embryo sac; G, gynoecium; I, integuments; M, microspores; N, nucleus; O, ovule; P, pollen; PMC, pollen mother cells; Se, sepals; Sg, stigmatic papillae; St, stamen; Sy = style; T, tapetum; V = vacuolated cell. (PDF 10 MB) [file 13227_2014_136_MOESM2_ESM.pdf]

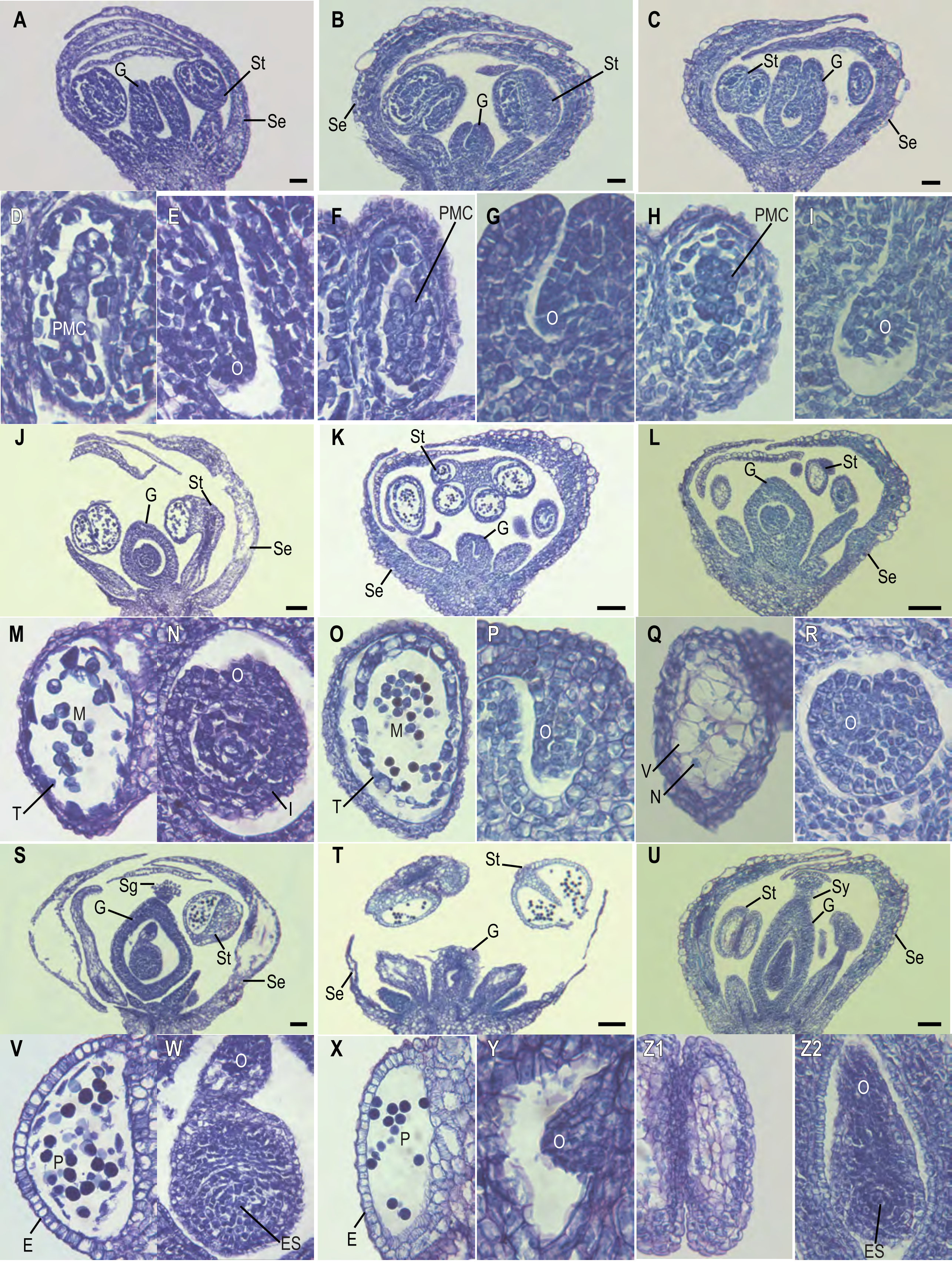

Additional file 2. Histological sections of hermaphroditic and dioecious *Lepidium* species stained with Toluidine Blue O. (A-C) Flowers at the pre-meiotic stage of microsporogenesis, stage 9; from left to right, (A) hermaphroditic *L. naufragorum*, (B) staminate *L. sisymbrioides*, and (C) carpellate *L. sisymbrioides*. (D, F, H) Anther locule from respective flower above. (E, G, I) Ovule from respective flower above. (J-L) Flowers later on in development, at the microgametogenesis stage, stages 11-12; from left to right, (J) hermaphroditic *L. naufragorum*, (K) staminate *L. sisymbrioides*, and (L) carpellate *L. sisymbrioides*. (M, O, Q) Anther locule from respective flower above. (N, P, R) Ovule from respective flower above. (S-U) Mature flowers, stage 13; from left to right, (S) hermaphroditic *L. naufragorum*, (T) staminate *L. sisymbrioides*, and (U) carpellate *L. sisymbrioides*. (V, X, Z1) Anther locule from respective flower above. (W, Y, Z2) Ovule from respective flower above. Scale bar = 50 μm in A-C; 100 μm in J-U. E, endothecium; ES, embryo sac; G, gynoecium; I, integuments; M, microspores; N, nucleus; O, ovule; P, pollen; PMC, pollen mother cells; Se, sepal; Sg, stigmatic papillae; St, stamen; Sy = style; T, tapetum; V = vacuolated cell.
